# Tailoring an intervention to the context and system redesign related to the intervention: A case study of implementing shared medical appointments for diabetes

**DOI:** 10.1186/1748-5908-3-34

**Published:** 2008-06-04

**Authors:** Susan R Kirsh, Renée H Lawrence, David C Aron

**Affiliations:** 1 Center for Quality Improvement Research, Louis Stokes Cleveland Veterans Affairs Medical Center, Cleveland, Ohio, USA; 2 School of Medicine, Case Western Reserve University, Cleveland, Ohio, USA

## Abstract

**Background:**

Incorporating shared medical appointments (SMAs) or group visits into clinical practice to improve care and increase efficiency has become a popular intervention, but the processes to implement and sustain them have not been well described. The purpose of this study was to describe the process of implementation of SMAs in the local context of a primary care clinic over time.

**Methods:**

The setting was a primary care clinic of an urban academic medical center of the Veterans Health Administration. We performed an in-depth case analysis utilizing both an innovations framework and a nested systems framework approach. This analysis helped organize and summarize implementation and sustainability issues, specifically: the pre-SMA local context; the processes of tailoring and implementation of the intervention; and the evolution and sustainability of the intervention and its context.

**Results:**

Both the improvement intervention and the local context co-adapted and evolved during implementation, ensuring sustainability. The most important promoting factors were the formation of a core team committed to quality and improvement, and the clinic leadership that was supported strongly by the team members. Tailoring had to also take into account key innovation-hindering factors, including limited resources (such as space), potential to alter longstanding patient-provider relationships, and organizational silos (disconnected groups) with core team members reporting to different supervisors.

**Conclusion:**

Although interventions must be designed to meet the needs of the sites in which they are implemented, specific guidance tailored to the practice environment was lacking. SMAs require complex changes that impact on care routines, collaborations, and various organizational levels. Although the SMA was not envisioned originally as a form of system redesign that would alter the context in which it was implemented, it became clear that tailoring the intervention alone would not ensure sustainability, and therefore adjustments to the system were required. The innovation necessitated reconfiguring some aspects of the primary care clinic itself and other services from which the patients and the team were derived. In addition, the relationships among different parts of the system were altered.

## Background

Successful implementation is a function of the relationship between the nature of the evidence, the context in which the proposed change is to be implemented, and the methods by which the change is facilitated [1,2]. However, operationalizing improvement within a specific context based on the literature is challenging, due in part to the limitations of the literature describing improvement efforts [3]. For example, often the rationale for the choice of an improvement intervention is not given except in the most general terms. Similarly, specific barriers, especially factors other than those related to the individual professional (*e.g*., factors related to the patient, the healthcare team, the healthcare organization and the healthcare system when addressed) also tend to be presented in general terms [4-14]. This lack of specificity inherently recognizes the need for decisions to tailor the general concept to the specific location, but at the same time there is rarely guidance provided for thinking about local challenges and opportunities. Nor is there guidance for making those types of decisions. This phenomenon of context-dependence has led to calls for tailoring interventions [6,15-20].

### Local and global problems

Although the concept of tailoring interventions is generally accepted, a systematic review of tailored strategies for behavior change in healthcare professionals revealed mixed results [20]. Moreover, much of the work describing tailored interventions has focused on individuals (end users), such as adaptation to patients' cultural background or adaptation of practice guidelines for healthcare professionals [21-24]. Thus, the process by which an organization-level practice change intervention can be individualized and implemented has not been well described. We suggest that part of the problem is conceptualizing the process as simply that of tailoring interventions to the context, and not recognizing or adjusting the unique local context to optimize success of that intervention. In fact, there have been relatively few studies of adaptation at multiple organizational levels, from the individual level (both patient and healthcare professional) to clinical microsystem, mesosystem, macrosystem, and even supramacrosystem. Even fewer describe the adaptation process itself, *i.e*., the basis for the choices made in determining the makeup of the intervention, and the evolution of the intervention over time. We will describe in detail the implementation of a specific intervention – shared medical appointments/group visits – in a specific context in order to elucidate these many issues.

### Intended Improvement

Shared medical appointments (SMAs) constitute a promising improvement strategy to help address the complexities and demands of managing chronic health conditions. There is evidence in support of this approach, including our own experience [25]. Shared medical appointments may also be called group visits, cluster visits, or chronic healthcare clinics. They have been described as a form of medical appointment with varying medical staff and patient populations and have been utilized for patients with chronic illnesses for whom education, self-management, and problem-solving skills are essential. The SMA is a patient medical appointment in which a multi-disciplinary team of providers (ranging from two to six) see a group of patients (eight to twenty) in a one and one-half to two hour visit. The implementation of SMAs was designed as a quality improvement project to improve intermediate outcome measures for diabetes – A1c, systolic blood pressure, low density lipoprotein cholesterol (LDL-cholesterol) – focusing on those patients at highest cardiovascular risk. We have previously reported the initial results in 44 patients who participated in these group visits: Levels of A1c, fell significantly post-intervention, with a mean (95% CI) decrease of A1c of 1.4% (0.8, 2.1) (p <0.001). The reduction in A1c was significantly greater in the intervention group relative to concurrent but non-randomized controls: 1.44 versus -0.30 (p = 0.002) [25].

While not all evaluations of outcomes associated with SMAs are as encouraging, the format remains appealing in an environment of growing demands and limited resources. In fact, the lack of success may be attributed to implementation challenges and issues that have not been adequately examined [26,27]. The general structure and processes for conducting SMAs have been established, but there is a lack of specific guidance to ensure success. As with other complex interventions, SMAs necessitate a systemic redesign that intersects a wide range of levels of a system (micro- to supramacro) for successful implementation and sustainability: SMAs require reconfiguring various levels of an organization's model of primary care.

### Study purpose

Our goal in this case study was to provide an in-depth analysis with the potential to identify themes and issues that will inform others interested in conducting or refining SMAs, or other organizational change. We describe the implementation and evolution of SMAs within a particular local context, a process that involved more than tailoring the intervention to the context; surprisingly, it also involved altering the context for intervention success. After initially considering the SMA as an addition to, or an enhancement of, the microsystem, we recognized over time that successful implementation required expansion of the clinical microsystem by creating an intra-meso structure within the constraints of the existing microsystem (one-on-one doctor-patient relationship) and mesosystem (primary care clinic) that is nested within a macrosystem (medical center) which in turn is nested within a supra-macrosystem, the Veterans Healthcare Administration (VHA) healthcare system.

## Methods

### Setting

This intervention was initiated within the primary care clinic of an urban academic medical center of the Veterans Health Administration. This clinic's primary care providers – five nurse practitioners (NPs), one physician assistant, eight part-time attending physicians, and 60 resident physicians – provide care for 11,000 patients, of whom 25% have diabetes. In addition to having a sophisticated electronic medical record, aspects of the Chronic Care Model routinely integrated into this clinic included nurse case management, and a clinical reminder system with feedback on performance [25]. The local context prior to initiating SMAs for patients with diabetes is outlined in Table 1, and follows a scheme adapted from Batalden *et al *[28]. The clinical microsystem is the small, frontline unit that is the primary clinical care unit (primary care provider and patient), which is nested within a mesosystem, and further nested within a macrosystem. Specifically, Table 1 defines the local context in January 2005 related to care for patients with diabetes and lists key elements related to diabetes care-based practices before introducing SMAs.

**Table 1:** Defining the local context prior to introducing shared medical appointments (SMAs)

**Care System Components**	**Defined via Local Diabetes Care Context**	**Existing Diabetes Care-Based Practices Pre-SMA (January 2005) **
***Supramacro***	VHA Central Office	Initiatives on outpatient quality with necessity to figure out how to operationalize locally
		Advanced Clinic Access mandate to reduce waiting times; increase efficiency
		Chronic Disease Index (a series of performance measures) emerging as a priority
		Electronic medical record tracking performance measures & providing feedback

***Macro ***	Cleveland Dept. of Veterans Affairs Medical Center	Pursue current mandate: Advanced Clinic Access to reduce waiting times for appointments
		Meetings about intermediate diabetes care goals
		Wanted updates about how goals were going to be met
		Primary care clinics focus on medical training not quality care
		Longer-term major construction creating space constraints

***Mesosystems***	Primary care clinics	Monthly reports about meeting diabetes care goals
		Monthly clinic meetings review & allocate resources
		No formal process to identify and refer high-risk patients
		Individual meetings with silo representatives
		Go to macro level for change if needed
	
	Other services	Primary care provider is additional signer on notes for patients
	
	Clinical pharmacy	Individual referral to education (meds and adherence)
	
		Medication algorithms (augment/adjust; problems)
	
	Health Psychologist	Referral to education: Medication adherence; barriers
	
	Nursing	Nurse manager meeting & viewed separately
	
	Clerks	Make appointments for follow-up/referrals
	
***Microsystems***	Individual Units	One-on-one meetings with patient
	
**Intra-micro**	~1,500 with A1c > 9%	Come for individual visits (every 3 months recommended)
**Patient**	High-risk	Follow-up with referrals to other services including:
		Pick-up new medications now and then see:
		Clinical pharmacist to change medications (1 month)
		Lab work prior to next visit
	
**Nurse**	2 Licensed practical nurses	Take vital signs, updates from patient, etc.
	4 Registered nurses	Provide case management/education as referred
	
**Provider**	**Primary care provider with diabetes patient:**	Expected to meet performance measures but limited support
		Worked individually with patient
	8 Part-time attendings	Goals A1c < 9%; LDL-cholesterol < 100 mg/dL; systolic blood pressure < 140 mmHg
	5 Nurse practitioners	Receive scores regarding % of patients meeting goals
	1 Physician assistant	If patient not meeting measures, then educate patient via:
	Preceptors (5 new)	Referrals for Consults to one or more (variable) specialists →
	Residents (60/year)	Nurse; Clinical Pharmacist; Nutritionist; Endocrinologist/Diabetologist
		Clinic; Health Psychologist ; Diabetes Self-management classes
		*Primary focus: medications to get to goal

### Planning the intervention

The microsystem prior to SMAs consisted of the patient care visit (primary care providers and patients). The visits consisted solely of one-on-one encounters with patients and differing providers (primary care provider, nurse, clinical pharmacist, and psychologist). The mesosystem was the whole primary care clinic where patients were seen. The clinic culture was characterized by a focus on individual responsibility of primary care providers rather than systems-based practice and there was relatively little interdisciplinary care. However, usual care also included referral to a dietician, certified diabetes educator, clinical pharmacist, or endocrine/diabetes specialty clinic at the discretion of the primary care provider. Thus, high-risk patients (part of the clinical microsystem) not meeting physiologic or process measure goals for diabetes were referred to any number of support staff for further education and treatment (mesosystem). A link back to the primary care provider existed via the electronic medical record. Additionally, different disciplines were not supervised by one director, but by leaders in their own discipline who did not work within the mesosystem. Changes in processes of care were difficult to achieve without many discussions with multiple discipline-specific supervisors. Improvement efforts previously were primarily top-down, based on mandates from the top management at the facility. At the macro-system level, the Cleveland VAMC was engaged in demonstrating quality measures for diabetes determined by the supramacro-system level of the VHA Central Office. At both supra and macro levels, there was increasing awareness of SMAs as a means to improve waiting times while meeting quality imperatives in an efficient manner. Organizational direction at the level of the macro- and supramacro-systems had a greater influence; there were mandates to conduct SMAs issued by the VHA, primarily to address issues related to waiting times and clinic access. Mandates from outside the local medical center aside, local leadership in general and in the primary care clinic in particular were strongly supportive of improvement efforts and open to the use of novel methods of care delivery. Moreover, the local facility has had a long history of support for and success in the implementation of clinical improvement allowing reliance on internal rather than external facilitation [29]. A committee formed to address the quality of diabetes care was an outgrowth of a day-long clinic retreat conducted off-site by two of the authors (SRK and DCA), among others. Clinic staff who previously had little involvement in system redesign began to take part.

### Planning the study

**Figure 1:**
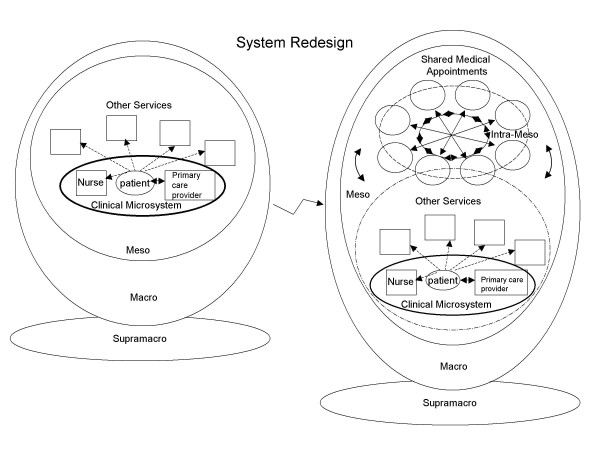
**Visual representation and framework for understanding the transformation (system redesign) associated with successful SMA implementation as intra-meso component. **The figure on the left side is the initial model and the right side includes the system redesign.

We used a nested systems framework to help organize and summarize implementation and sustainability issues [28]. Figure 1 provides a visual representation and framework for understanding the system redesign associated with successfully tailoring the intervention and the local context. Specifically, the left side of Figure 1 depicts the initial conceptual model of our healthcare system. The microsystem links to the mesosystem in that patients are referred, as needed, to nursing and other services. The macrosystem level refers to the local organization. The local organization is also linked to the national organization (supramacro level). We represented the supramacro-system as a perpendicular layer to emphasize the role as a foundation and the distant though defining influence of the supramacrosystem on the local context. Figure 1 also depicts the conceptual model that evolved to describe the successful implementation of SMAs for patients with diabetes (right side of the figure; see below for discussion).

### Methods of evaluation and analysis

We used an in-depth case analysis approach focusing on the context and methods of implementation. This allowed us to describe the conceptual issues related to system redesign to implement an SMA for patients with diabetes [1,2,30-35]. In particular, we used the characteristics of innovations framework of Grol *et al*. to characterize SMAs as an innovation in terms of the factors that might promote or hinder implementation processes [32]. We have used a participatory/action research approach where relevant parties of the process actively examine, plan, evaluate, and reflect throughout the cycles. [36] Such an approach best achieved our goals related to capturing the processes and key elements impacting on those processes. [37-40]. Notes from meetings and debriefings, including feedback from primary care providers, patient surveys, e-mails, and meeting summaries (*e.g*., meetings of the team developing the related research grant application and the practice manual), were reviewed, cataloged, and coded for relevance to the implementation process. In addition, this quality improvement project took place in concert with the Academic Chronic Care Collaborative sponsored by the Association of American Medical Colleges and the Institute for Healthcare Improvement. Monthly reports submitted to this Collaborative were reviewed. This was done in an iterative process combined with interviews with key participants and observations. Seven local individuals familiar with the implementation processes of this project or SMAs were asked to independently review the summaries and findings. Six provided written feedback and were interviewed in a semi-structured format for validation purposes. The model presented here and the formats for structuring the presentation emerged from this participatory/action based and grounded-theory approaches [36,41].

## Results

### Accommodating the innovation into the local context: initial decisions

Once the decision was made to begin SMAs, it was necessary to create general guidelines about SMAs and translate those into the local context, with its resources and needs. Implementation fidelity is often presented as critical to achieving the levels of efficacy demonstrated in clinical trials. However, it became apparent that descriptions of SMA interventions provided insufficient detail to guide implementation into differing clinical settings. While decisions and potential options were sometimes discussed, guidance on translating and mapping out to the local context was not provided. Table 2 outlines the initial dimensions of the SMA innovation we identified (first column). The second column delineates our initial decisions or translation of the intervention to the needs of the local context. In order to maximize success and meet demanding clinical care needs, we began with diabetes as a focus because of the existing core team and its openness to change, some collaboration between key disciplines was loosely in place, the volume of patients with diabetes, the cost to the organization, and the high demand of resources required to manage patients with diabetes. However, as is true with most decisions, there were aspects of many decisions that included promoting factors but also came with hindering factors. Therefore, Table 2 also outlines the promoting and hindering factors associated with each of the initial decisions.

**Table 2:** Analysis of SMAs Innovation: Translating SMA into Local Context (February 2005)

**Dimension of SMA Innovation – Basic guidelines that needed to be translated**	**Starting Point: Initial Decisions **	**Promoting Factor**	**Hindering Factor**
**Shared Medical Appointment Initiation**	Core team with strengths related to diabetes were open to change and working together	Mandate from Central Office; Training provided; no specific guidelines; local facility has long history of supporting novel methods of care delivery	No specific guidelines; limited resources

**Focus**: disease-specific or non-specific	Diabetes (reduce cardiovascular risk)	Provided focus consistent with strong core team	

**Drop-in or Schedule Patients**	Scheduled	Able to call and remind; able to plan	Limits number and requires more coordination

**Multi-disciplinary Professional Team**	Collaboration with key disciplines present	Strong, committed core team, including one member representing key leadership within primary care clinic	Difficulty coordinating, and finding and freeing up time to participate

1 or more with prescribing Authority	Physician (Medical Director of Clinic); Endocrine nurse practitioner; Clinical pharmacist	Built-in redundancy of prescribers assisted with efficiency	Team members had different supervisors; Workload credit and credit for SMAs

1 or more variety of Disciplines	Health Psychologist; Registered nurse		Different supervisors; Workload credit

Group of patients (8–20)	4–8 patients (8 invited)	Flexibility to pilot test with small numbers of patients	Questions raised about inefficiency

**Target population**	Local registry to identify patients	Sufficient numbers who would benefit	

Primary care provider pool (pull from one or more)	All Primary care providers' patients eligible	Able to include all high- risk patients	Threatened provider-patient relationship

Patient pool	A1c > 9%; systolic blood pressure > 130 mmHg; LDL-cholesterol > 100 mg/dL		Getting several patients there; Viewed as difficult and non-compliant; concern about no-show rates

**Time and Frequency: **Meet for 90–120 minutes and variable regarding frequency	90 minutes and to meet weekly (Friday afternoons)		

**Techniques and Processes for conducting SMA**	Modification of chronic care model as a guide		

Didactics	Keep at a minimum		Many team members most comfortable with 'teaching' rather than facilitating group discussion

Information display and Sharing	Large board with patient lab values and other outcomes (*e*.*g*., A1c, systolic blood pressure and LDL-cholesterol); prepared by Clinical pharmacists	Summarized key points and helped solidify take home messages despite concern about non-lecture format	

Group discussion	Peer support Motivational interviewing by Health Psychologist	Learning by all is possible even if not sharing; Simplified and focused individual session that followed group encounter	Some patients uncomfortable in groups

Clinical component	Group chart display		

Forms: General information	ABCs of diabetes care (A1c, blood pressure, cholesterol, etc), foot care, etc.	Able to help meet performance measures; document patients educated	Hard to clarify for others what exactly was covered

Forms: Patient-specific	Patient completed form with current values (copied from board), goals, med changes, plan of care outlined	Felt patients were getting individual information and tailoring	Preparation time

**Space**	Remote training rooms not available and negotiated clinic space	Able to secure some space	Limited options especially given construction

Location	Primary Care Clinic Conference Room	Familiar	Displaced providers who use the room and limited access to computers available in the primary care clinic conference room

Size and arrangement	Small conference room with computers and crowded	Table seating conducive to group sharing	Limited in size and mobility; configuration not ideal

**Mechanics**			

Documentation (suggest/identify individual to take responsibility)	Initially used a group note field in electronic record system, but recognized that modifications would need to be made.^1^	User friendly, consistent with usual methods of documenting	

^1^The group note fieldallows text to be entered that will appear in the note of every patient in the group. However, it was recognized early on that such a note did not allow for customization. Therefore, we initiated the development of a templated note with embedded guidelines that was user-friendly and facilitated the efficiency of documentation and standardization and completeness of individual treatment plans. This development took place over a period of several months.

It is worth highlighting key promoting factors for the innovation that relate to the system levels because ultimate system redesign requires successful alignment and interplay between all levels. While the organizational structure is very hierarchical (Figure 1), there was openness to novelty. In fact, there was the supramacrosystem level mandate to begin SMAs, with considerable latitude given to how those mandates were achieved. Descriptions of the transformation of the VHA describe these seemingly contradictory strains [42]. Thus, at the supramacrosystem level, promoting factors included the mandate for action to address performance deficiencies, the so-called 'burning platform' and the simultaneous freedom and flexibility to pilot test to secure buy-in [43]. At the macrosystem level, there was similar support for innovation. At the mesosystem level, a strong core care team was essential that reflected multi-disciplinary members from the various services that would be linked. This team was open to new care models and expanding roles with a leader who had the ability to make changes at the microsystem level. Although Table 2 identifies a number of promoting factors, we believe that the most essential factors were the formation of a core team committed to quality and improvement, and the leadership provided by the clinic director that was supported strongly by the team members.

At the same time, there were several key innovation-hindering factors associated with the general mandate to conduct SMAs and the specific decision to translate the mandated innovation into the local context: limited resources (such as space); potential to alter longstanding patient-provider relationships; organizational silos (disconnected groups) with core team members reporting to different supervisors; difficulties in documenting workload for credit; and finally, the flexibility itself and absence of specific guidelines for meeting the mandates, resulting in a certain inefficiency and delay in the process. Implementation in a space-constrained facility that was in the midst of major construction and renovation meant that the choice of a location resulted in displaced providers who used the space, and limited access to computers available in the conference room. There was concern that group visits with different providers would disrupt established provider-patient relationships and inhibit those providers from referring patients. The different lines of authority for each of the core team members necessitated negotiations with four different supervisors, some of whom were more open to SMAs than others. In this organization, there is a strongly perceived need (varying among different clinical and administrative departments) for meticulous accounting of one's workload. It was not intuitively obvious how to account for SMA work within current accounting systems.

### Implementation and evolution

SMAs require complex changes that impact on care routines, collaborations, and various levels of the organization. As such, implementing the initial decisions involved more than putting decisions into place. As noted by others, implementers and champions of innovation are critical. This is particularly true the more complex the change and the need for system redesign. Those who conduct and carry out the implementation obviously play a key role in helping to initiate and sustain the intervention. Implementers for our SMA intervention included a physician who was the Medical Director of the clinic and an Endocrine Nurse Practitioner. The physician was an established leader of the Primary Care Clinic for two years prior to initiating the intervention and had some training in Quality Improvement. The physician felt ownership of the improvement processes overall and had the authority to solicit and get approval for staff in other disciplines to participate in the SMA. The Endocrine Nurse Practitioner was not a member of the Primary Care Clinic but was considered to be a content expert and opinion leader at our institution. She had worked with high-risk patients with diabetes for 20 years prior to the intervention and was willing to share her expertise with patients as well as other less knowledgeable team members. All members of the core team were strongly committed to working together and were key stakeholders at the mesosystem level.

Although the initial analysis and translation of the innovation (Table 2) provided a starting point and the implementers provided additional local motivation, further analysis of the SMA beyond the promoting and hindering factors associated with the decision to implement was necessary for guidance to tailor and adjust the innovation to the local context. Grol *et al*. identified a series of characteristics of innovations that might promote or hinder implementation processes [32]. The relationship between these factors and the local context is outlined in Table 3. While the relative advantage/utility was appreciated by the initiators early on, three other innovation characteristics also appeared to be critical to successful implementation: compatibility, involvement, and collective action. This innovation was very compatible with the norms and values of the institution in promoting improvement in chronic disease quality measures. The involvement of the core team who would be implementing the SMA was very high. Individuals met to collectively decide the specific details of the clinical experience for patients and providers. However, hindering factors included: low compatibility with the traditional one-on-one visit with a primary care provider, high complexity in that the innovation was difficult to explain, and low collective action from the primary care providers who did not have input into the SMAs into which their patients would be recruited.

**Table 3:** Key implementation and evolution factors using Grol and Wensing's Characteristics of Innovations Framework [32].

**Characteristic of Innovation ~Degree to which innovation provides or is: **	**Promoting Factor for SMA Implementation**	**Hindering Factor for SMA Implementation**	**Addressing the Issues to Facilitate Implementation and Sustainability**
**Relative advantage or utility **over existing or other methods	Advantage of seeing several experts at same time, especially for behavioral barriers	No clear evidence; questioned value and whether patients would accept group format	Proved not to be a major issue

**Compatibility **with existing norms and values	Consistent with norm and values of achieving process measures	Inconsistent with norm and value of sacred primary care provider-patient relationship; Different roles of healthcare professionals filling in-difficult switching from traditional to multidisciplinary team approach	Had a few team building and motivational interviewing learning sessions-lecture versus facilitation of patient info

**Complexity **of explaining, understanding and using		Too vague and many unknowns; not easy to explain	Explain and sell it and take advantage of a trial period with small numbers of patients to highlight success and have observers (it was easier for providers to see it first hand)

**Costs **relative to benefits and level of investment		Efficacy questioned regarding clinical physiological outcomes and uncertain level of investment for various stakeholders	1. Reorganizing flow allowed up to 18 patients to be seen in one SMA
			2. Change in way patient data distributed in order to reduce prep time of Clinical Pharmacist and overall cost
			3. Introduced use of templated notes that included documentation of SMA activities at a general group level and also permitted individualized patient level documentation

**Risks **related to uncertainty regarding results and consequences		High-risk – no conceptual model for designing or plan for diffusion	The organizational culture supported risk taking

**Flexibility, adaptability **to situation/needs of local context/target group	Vagueness provided options for adapting to local context and needs	Key non-flexible components not consistent with micro-system and mesosystem silo design	Recognition of additional patient needs prompted addition of a nutritionist to the team

**Involvement **of target group in development	High involvement of the core team only	Existing structure impeding additional staff involvement	Unanticipated impact on staff not involved feeling left out addressed by creating opportunities for these staff to observe and get feedback/up dates

**Divisibility **so able to try out parts separately		Low divisibility of shared appointments (*i*.*e*., can't try out various parts)	Unable to address; we have kept the basic model of SMAs

**Trialability, reversibility **without risk if doesn't work	High and approached as a trial period		Because of early successes, this proved not to be a major issue

**Visibility, observability **of results by other people	High – part of local culture is feedback	High – part of local culture is feedback	Patient successes led to increased referral of patients close to performance measure goals overloading the clinic and prompting the redirection of resources

**Centrality **of impact on daily working routine		High	Impact of patients' stories has contributed to team finding meaning in their work, negating the effects of the changes in work routine

**Pervasiveness, scope, impact **on total work, people involved, time it takes and relationships		High: fear more work and would jeopardize primary care provider-patient relationships	Proved not to be a major issue

**Magnitude, disruptiveness, radicalness**		High	The core team was made up of individuals willing to take risk and were unafraid of the potential disruption

**Duration **for when innovation/change must take place	Not a pressing factor		

**Form, physical properties **of innovation: material or social; technical or administrative, etc.)		High: material change, space requirements, schedule changes, administrative and technical adjustments	Continues to provide challenges

**Collective action **related to decisions	Low collective action	Strong core team (3–5 members)	Unanticipated impact on staff not involved feeling left out. Some of these staff were recruited to participate in other types of SMAs where they were involved in the decision-making.

***Nature of Presentation***: length, clarity, attractiveness	High attractiveness	Low clarity	Began projects to share knowledge and experience with others

The initial decisions and implementation endeavors began the process of practice change, but iterations of tailoring the intervention and negotiating system redesign were necessary. While not surprising that there would be issues on the path from start-up to sustainability, little attention has been given to identifying and categorizing them. Within our local context, the SMA process for patients with diabetes has changed over the last two years. These changes have occurred at the level of the clinical microsystem, mesosystem, and macrosystem. Within the microsystem, many changes have involved team structure, the patient population, and clinic flow. In Table 3, we have used the Grol *et al*. framework to list the key changes over time and strategies for promoting implementation and sustainability [32]. This framework identifies the flexibility and adaptability during implementation as a dimension which can either promote or hinder the process. We found that because our SMA had a strong core team, this was an important aspect to identify and maximize throughout implementation. Once identified, we could use this promoting factor to offset challenges encountered during implementation. The lack of clear designation of what the innovation and team members needed permitted the team to adapt the innovation to the local context and needs throughout the implementation process. As an example, we recognized after initiation of the SMA process that patients wanted to discuss dietary issues in detail, and we subsequently added a nutritionist. Another example is the response to the challenges of documenting the patient visit. We initially used the group note function in our electronic medical record. The group note field allows text to be entered that will appear in the note of every patient in the group. However, it was recognized early on that such a note did not allow for customization. Therefore, we initiated the development of a template note with embedded guidelines that was user-friendly and facilitated the efficiency of documentation and standardization and completeness of individual treatment plans. This development took place over a period of several months. Another characteristic is that of complexity of both the innovation (SMA) and its implementation. The SMA was something that was identified initially as a vague unknown type of clinical care which was not easy to explain to the primary care staff. This constituted a barrier to successful implementation. We decided to take advantage of a trial period with small numbers of patients to highlight success as well as allow clinic practitioners to sit in on one to three SMAs. Through identification of this barrier we were able to develop a strategy to overcome it.

### Results: Evolution of the conceptual model

The right side of Figure 1 depicts the conceptual model that evolved with the successful implementation of SMAs for patients with diabetes. The system redesign that resulted from implementing SMAs included continuous tailoring of the intervention to and continuous adjustment of the local context. This interplay of co-evolving components added a new clinical venue to which referral of patients was possible. SMAs were designed with the idea that they would exhibit the characteristics of a high-performing clinical microsystem; *e.g*., alignment of roles and training for efficiency and staff satisfaction; interdependence of the care team to meet patient needs; integration of information and technology into work flows; and supportiveness of the larger organization [36,44]. However, we felt that to conceptualize SMAs as another clinical microsystem was confusing, given the co-presence of the more traditional microsystem and the unique way SMAs expanded and integrated other services and resources of the primary care clinics that was contrary to traditional thinking about care. Moreover, the primary responsibility for the patients seen in the SMAs was and would remain in the hands of the primary care provider in his or her microsystem. Accordingly, SMAs are identified as an intra-mesosystem component to recognize the linkages among and between other meso components (intra-meso) beyond the microsystem, and to emphasize the system redesign. Additionally, the SMA with its own iterative improvements and evolution seemed a separate system as opposed to a higher functioning system that already existed. This is in contrast to the initial system design where there was only the closed microsystem with the components within (intra-micro) the inner clinical microsystem.

System redesign is also reflected in the arrangement of the SMAs: the squares in Figure 1 represent participants on equal footing by recognizing the role of each discipline's expertise, including the patients who also bring expertise to the exchange. In addition, the graphic representation of the flow of communication underscores the mutual contributions and simultaneous, non-sequential nature of the interactions for patients and providers. Finally, the clinical microsystem and the intra-mesosystem (SMAs) are overlapping to reflect that SMAs do not eliminate the traditional clinical microsystem but rather offer another opportunity for care, with both approaches co-evolving. This point is particularly important to recognize, as one concern providers often expressed was the potential undermining impact SMAs might have on the individual provider-patient relationship.

### Local context and sustainability of SMAs two years later

**Figure 2:**
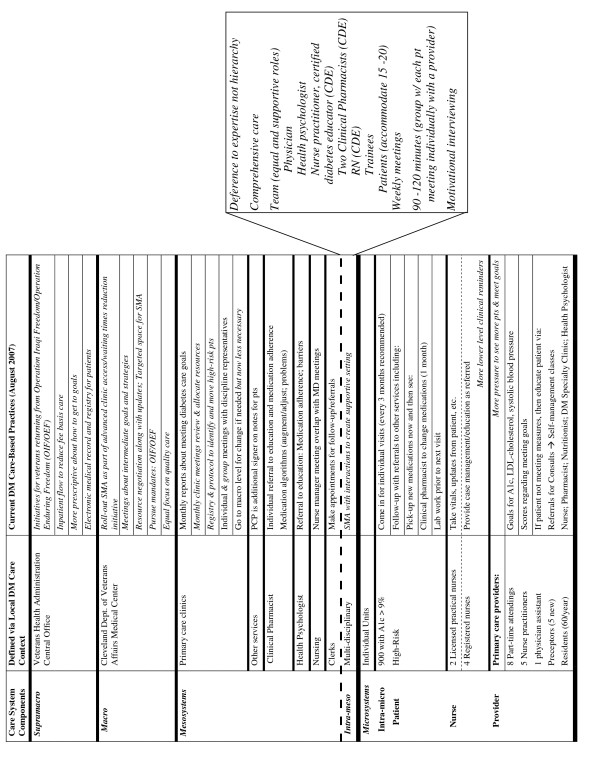
Current (post-transformation) local context and care-based practices related to diabetes management.

The current local context and care-based practices related to diabetes are summarized in Figure 2. Changes or differences are denoted in italics, with items directly impacting on diabetes care aligned on the right side of the last column. The current state of the SMAs for patients with diabetes is summarized in the pull-out box that reflects the intra-mesosystem redesign level. Figures 1 and 2 help to identify the major changes and shifts in local context as well as the issues related to tailoring the intervention and adjusting the context.

It is worth highlighting some issues at each level of the care system. At the supramacro level, while continued improvement in information technology helps further support the SMA as configured at the local level, the mandates and priorities have changed. While this is to be expected, it does alert innovators and implementers to appreciating windows of opportunity. If the innovation has not taken off and achieved a force of its own (including demonstrating some levels or areas of success consistent with the organizational goals), changing priorities (new mandates), and the lack of success will create increasingly difficult challenges.

Given the demonstrated successes, leaders at the macrosystem system want the SMAs to be expanded to other conditions and possibly other care sites, *e.g*., the community-based outpatient clinics linked to the main facility. Some new or adjusted practices beyond the actual SMA venue at the mesosystem level have also come about because of SMAs (*e.g*., monthly clinic meetings to discuss resource allocation and group meetings among discipline representatives) and some will help to further propel SMAs forward (*e.g*., registry and protocol development to identify high-risk patients).

At the microsystem level, primary care providers are experiencing more pressure to meet performance measures of quality and productivity (and at the supramacrosystem level, the current context is also for more prescriptive approaches about how to achieve goals). The objectives of the diabetes SMA map out to the increased pressures experienced by providers. Seeing the successes of the SMA, providers began to send patients with A1c levels very close to goal. This was not necessarily all positive, as we were unable to accommodate those identified in the registry with an A1c of greater than 9%. While the magnitude of the increase in referrals to SMAs created some unanticipated adjustments, we have worked and continue to work and negotiate with providers to prioritize resources. Their clear desire to refer more patients to SMAs underscores the growing foundation for sustainability.

Many factors contribute to implementation and sustainability of the SMA within the mesosystem (intra-mesosystem component) and with regard to its relationship to the clinical microsystems. Most importantly is how the SMA is valued. The increased number of referrals is evidence of the value placed on SMAs by the mesosytem providers. SMAs are valued by the professionals on the team based on their experiences with patients and on their feelings of a high degree of 'teamness', or esprit de corps [45]. Team members meet after each SMA where various members take turns working a little extra to support the SMA during non-clinical time with activities like making extra phone calls, generating letters to new patients, tracking patient satisfaction data, and meeting to change flow, if needed. In addition, the flexibility of the individual team members is manifest during the SMA sessions; all staff members pitch in with clerical duties as needed, re-check blood pressures, and download glucometers. A weekly meeting after each SMA continues to occur to discuss patients and processes to assure that all team members have an open forum to voice concerns and make group changes, thus maintaining the high degree of shared governance. In addition, beyond improved clinical outcomes, patient satisfaction has helped confirm the added value to providers and to administration (macro- and supramacrosystems). Patient satisfaction surveys routinely are administered following the SMA. Typical comments from patients have included: 'I learned a lot', 'this clinic really takes such good care of patients' and 'I wish this kind of clinic existed 20 years ago.'

## Discussion

Although the SMA originally was not envisioned as a form of system redesign that would alter the context in which it was implemented, it became clear that tailoring the intervention alone would not ensure sustainability without adjustments to the system. The innovation necessitated reconfiguring some aspects of the mesosytem (the primary care clinic and other services from which the patients and the team were derived) and relationships with the microsystems. Though not specifically anticipated, evolution of the former was associated with evolution of the latter: As the patients from the primary care providers were seen in the SMA and then transitioned back to them there was transfer (spread) of management knowledge in patients with diabetes, particularly among the nurse practitioner providers. We have gained the impression that many more primary care providers initiate insulin appropriately at a lower threshold. Additionally, the use of patient check-out sheets that had been modified for the SMA to include lab results, targets, and self-management goals have been adopted by primary care providers. When they reviewed the SMA sheets, they wanted the additional information on the check-out sheets for all clinic patients, not just patients seen in the groups.

Other sustainability ingredients observed at the mesosystem level include a high level of interest and volunteers to help staff additional SMAs for hypertension and hyperlipidemia. This will provide the cadre of individuals necessary for sustainability. At the macrosystem level, the implementation of a SMA for diabetes and its success has come to the attention of the Chief of Staff and Medical Center Director. They have committed resources (*i.e*., financial resources) to maintain our clinical registry which serves as part of the critical infrastructure supporting SMAs. There has also been some redesign of clinic space to better accommodate the needs of SMAs. A construction project is planned to create a new room within the clinic designed specifically for group visits.

The fact that implementation could be accomplished without requiring the hiring of additional personnel facilitated gaining administrative support (macrosystem), but this required a compromise – using a less cost-effective approach to visit documentation. The organizational mandate and sponsorship of the clinic leader allowed time to be freed up for the staff. Different policies had to be developed for scheduling for the new clinic. Over time, this innovation became 'accepted practice' and SMAs for other conditions have been established; the mesosystem redesign factors and processes directly and indirectly related to SMAs for patients with diabetes is ongoing. Recent analyses of routine clinical quality assurance data have suggested the continued beneficial patient impact of SMAs.

## Limitations

Our study has a number of limitations. First, this was a retrospective study of a quality improvement project. Standardized written materials pertaining to the implementation were limited. Consequently, recall of information with its attendant shortcomings was an important source of the data. However, all available material was evaluated systematically and triangulated with other individuals. Second, the study is limited to one local context and one intervention. At the same time, the issues faced – both the challenges and opportunities – are not unique. While predicting change and its course are challenging, understanding case studies of the process of individualizing or tailoring interventions to the existing and evolving environment provides important lessons, as others have noted [46]. Our adaptations did not sacrifice the improved clinical outcomes, and in fact may have enhanced clinical outcomes. Thus, our study provides insights into a successful implementation process for SMAs by describing how we addressed initial contextual decisions, and why those decisions were made, by identifying factors and considerations that necessitated adjusting initial decisions. Actions regarding issues ranged from clinic set-up to provider roles and tasks, sharing strategies to sustain this clinic as an adjunct to the primary care clinic, and providing information on how and what we chose to evaluate.

## Conclusion

Our study describes in detail the processes by which an improvement intervention and the local context co-adapted and evolved during implementation, and continue to evolve to ensure sustainability. In theory, high implementation fidelity is necessary to achieve the levels of efficacy demonstrated in clinical trials. However, it is also clear that interventions must be tailored to meet the needs of the sites in which they are implemented [19,20,23,47]. Unfortunately, guidance on specifics of that tailoring to the practice environment is lacking. We have described initial key characteristics of the intervention (SMA) that required adaptation and those perceived important to maintain without change if successful implementation was to occur. By identifying innovation characteristics as they pertain to our innovation (SMA), we illustrate the iterative processes involved in implementation [32]. Although specific factors of the intervention and the context appeared to be critical in this particular circumstance, it is most noteworthy that both the intervention and the context (at multiple levels) had to change. By illustrating key portions, we found that the more the intervention necessitated organizational and structural changes the more difficult it was to translate successfully. A strong core team, clinical improvements and an organization that supports innovation were paramount in overcoming the inertia inherent in clinic structure redesign, a necessary step to sustain the innovation. This system redesign needed to happen and could not have occurred devoid of appreciating and co-adapting the context and the intervention. It is also imperative to recognize the iterative nature of successful implementation that occurred as a part of our evolution. This iterative practice re-evaluation has now become manifest in other SMAs implemented (heart failure and hypertension), thus continuous quality improvement has become ingrained into routine operation in SMAs. This differs from many of the other clinics. Although clinical improvements have been sustained for two years, it is also important to recognize that outcomes need to be assessed at multiple levels – patients, staff and organization. For staff, this could include satisfaction as well as knowledge and skills. For the organization, it could include cost and efficiency as well as organizational climate and culture. A comprehensive set of measures would constitute the balanced scorecard necessary for overall system optimization.

## Competing interests

The authors declare that they have no competing interests.

## Authors' contributions

The authors shared equally in the conception of the study, design, coordination and drafting the manuscript. All authors work in the medical center with two working in the clinic (SRK and DCA). Specifically, two of the authors (SRK and DCA) participated in the initiation and development of the SMA, with SRK being a regular team member and DCA being a back-up team member. All authors read and approved the final manuscript.
